# Correction: Niche partitioning facilitates coexistence of closely related honey bee gut bacteria

**DOI:** 10.7554/eLife.78825

**Published:** 2022-04-01

**Authors:** Silvia Brochet, Andrew Quinn, Ruben AT Mars, Nicolas Neuschwander, Uwe Sauer, Philipp Engel

**Keywords:** Other

Brochet S, Quinn A, Mars RAT, Neuschwander N, Sauer U, Engel P. 2021. Niche partitioning facilitates coexistence of closely related honey bee gut bacteria. *eLife* **10**:e68583. doi: 10.7554/eLife.68583.

Published 19 July 2021

After the publication of the article we realised that there was an error in the labelling of the y axis of several panels of Figure 2 and Figure 2—figure supplement 2. As these figures refer to in vitro and not the in vivo experiments, the y axis should say “ml” (of culture) instead of “gut”. In addition, we realised that it would be more appropriate to use the term “bacteria/gut” and “bacteria/ml” instead of the current “read counts/gut” for the y axis of the panels containing amplicon sequencing data normalised by CFUs/gut or CFUs/ml. We have made the following corrections:

Figure 1: Labelling of y axis of panels B. and C.: bacteria/gut.

Figure 2: Labelling of y axis of panels A., B., C. and E.: bacteria/ml.

Figure 2—figure supplement 2: Labelling of y axis of panels A., B. and C.: bacteria/ml.

The corrected Figure 1 is shown here:

**Figure 1:**
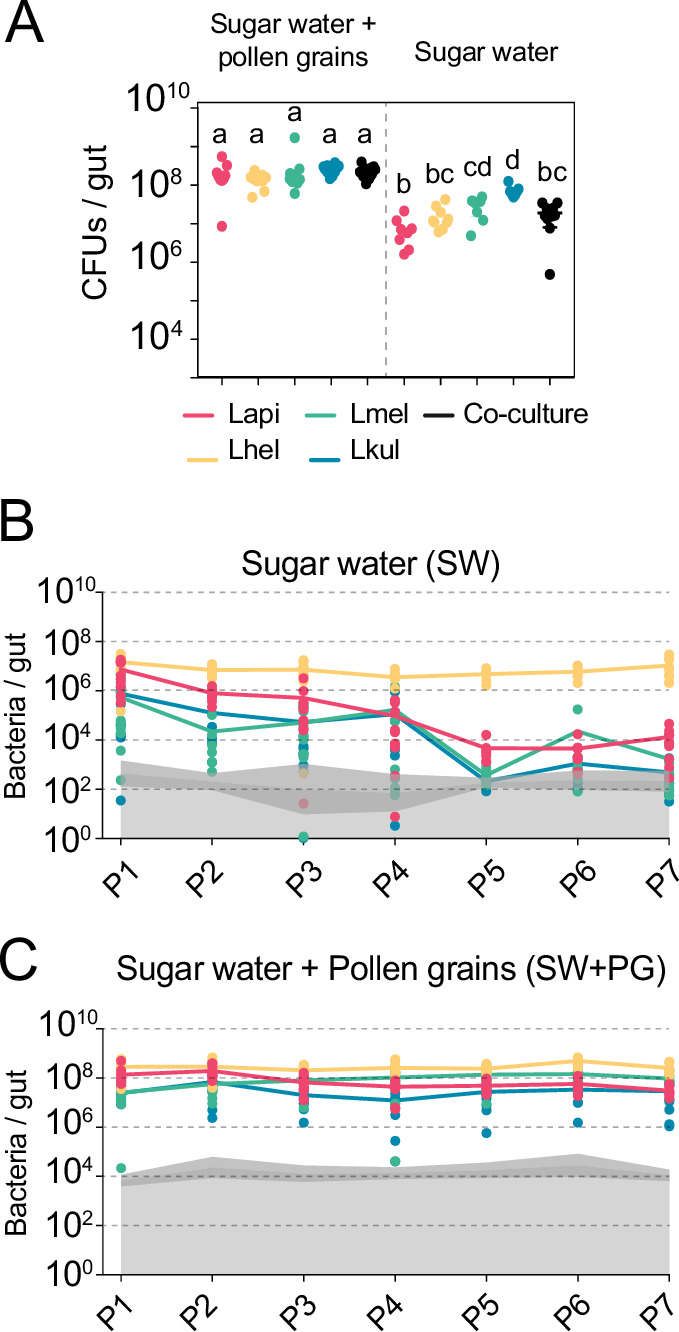


The originally published Figure 1 is shown here for reference:

**Figure 2:**
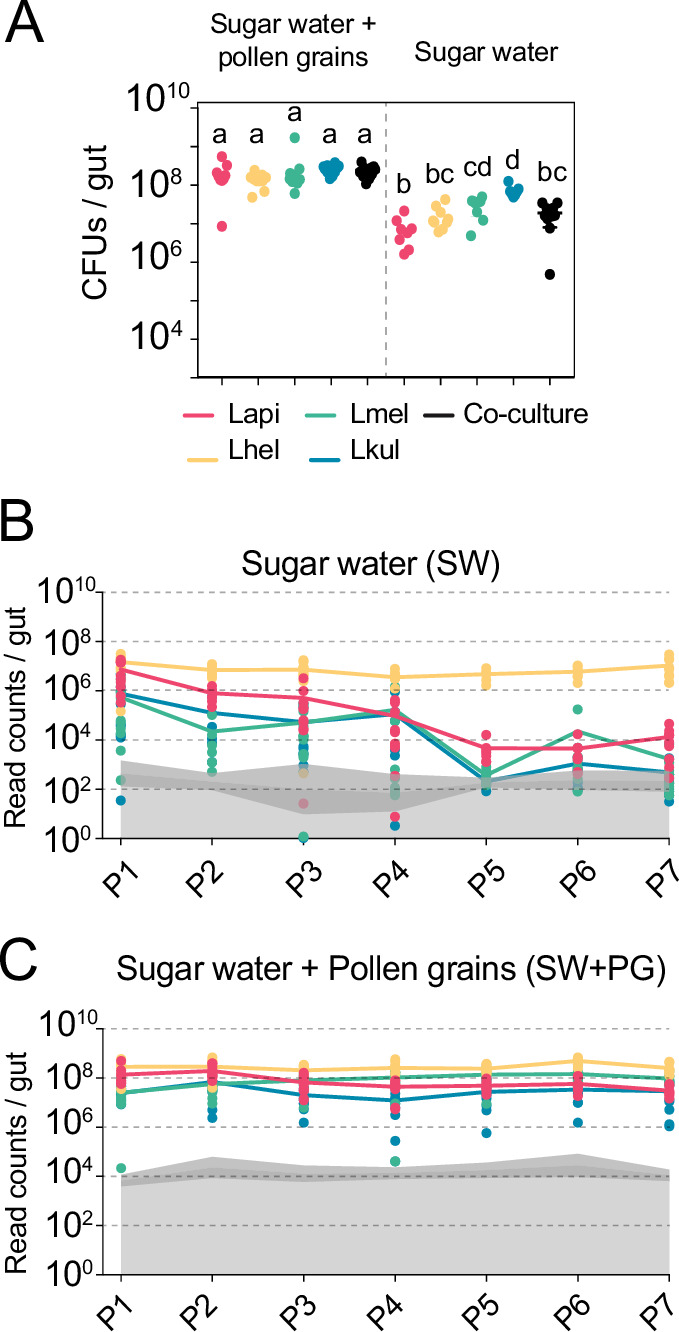


The corrected Figure 2 is shown here:

**Figure 3:**
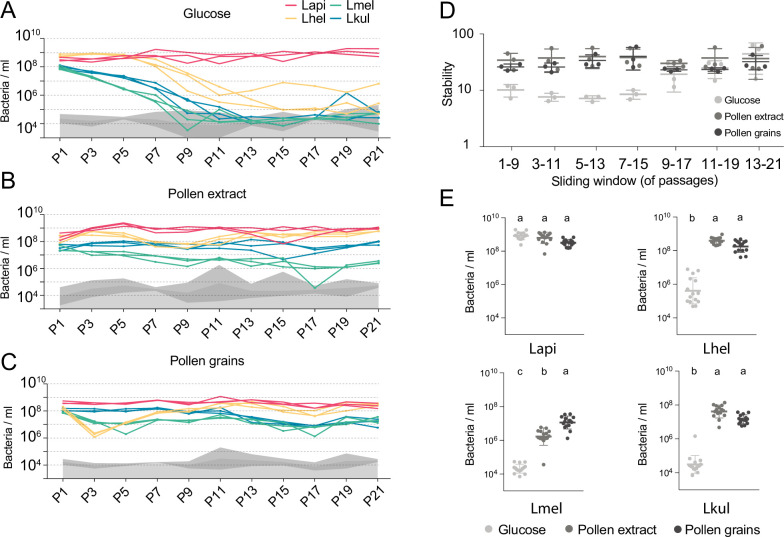


The originally published Figure 2 is shown here for reference:

**Figure 4:**
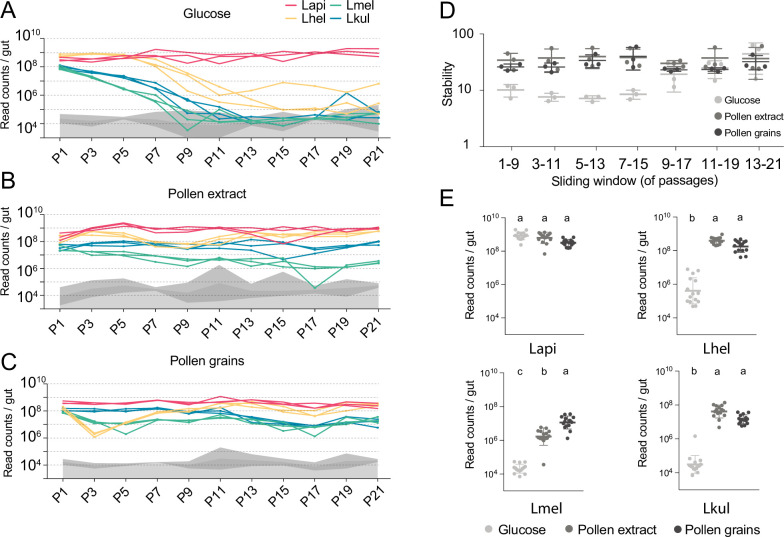


The corrected Figure 2—figure supplement 2 is shown here:

**Figure 5:**
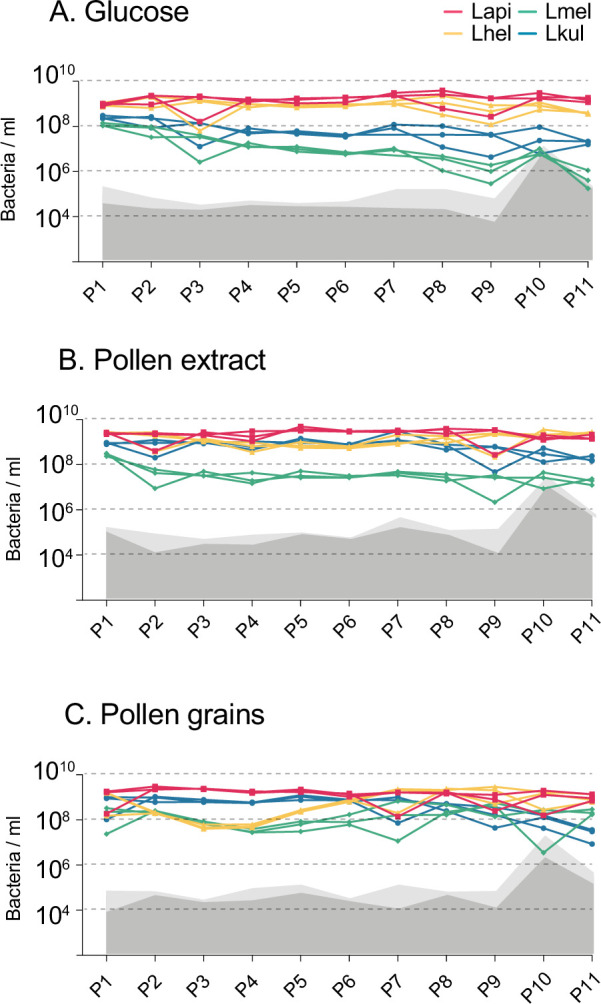


The originally published Figure 2—figure supplement 2 is shown here for reference:

**Figure 6:**
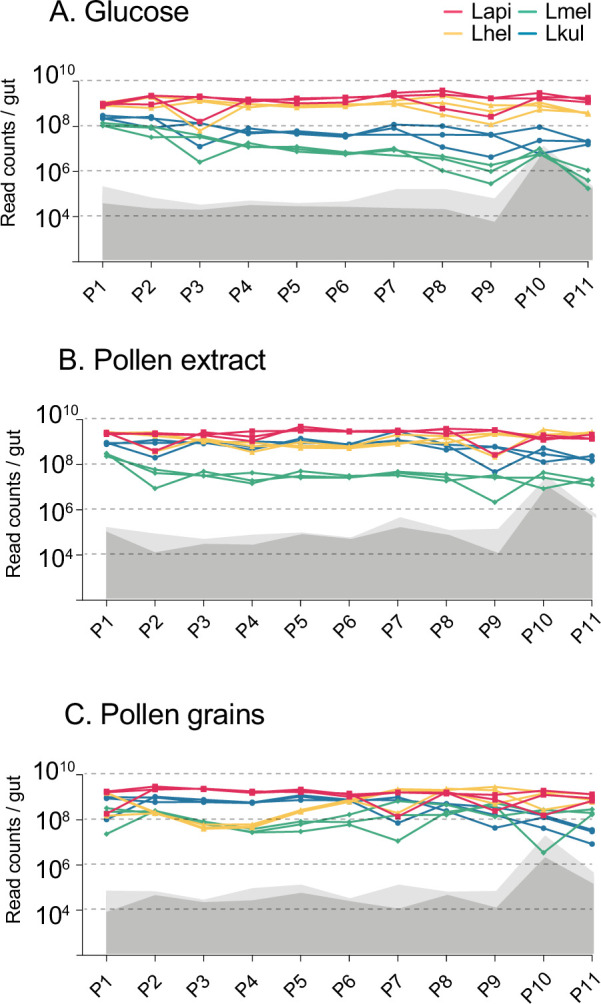


These labelling errors do not affect the results and conclusions of the original article.

