# Supplementary material for: Niche partitioning facilitates coexistence of closely related honey bee gut bacteria
Source: eLife. 2021 Jul 19;10:e68583. doi: 10.7554/eLife.68583 (PMC8456714; doi:10.7554/eLife.68583)
Supplement: Supplementary file 1. [file elife-68583-supp1.docx]

**Amplicon Sequencing PCR I:**

| **F**: CGTACGTAGACGGCCAGTATGCCNGAAATGCCRGARGTTGA |
| --- |
| **R**: GACTGACTGCCTATGACGACTAARCGATAYTTRCCYTCCATRCG |

**Amplicon Sequencing PCR II:**

| **B1F/B1R** | CAAGCAGAAGACGGCATACGAGATTAAGAGGCGTCTCGTGGGCTCGGAGATGTGTATAAGAGACAGATACGTACGTAGACGGCCAGT/AATGATACGGCGACCACCGAGATCTACACGCCTCTTATCGTCGGCAGCGTCAGATGTGTATAAGAGACAGGACTGACTGCCTATGACG |
| --- | --- |
| **B2F/B2R** | CAAGCAGAAGACGGCATACGAGATGCGAATTCGTCTCGTGGGCTCGGAGATGTGTATAAGAGACAGTACGTACGTAGACGGCCAGT/AATGATACGGCGACCACCGAGATCTACACCAGCGTATTCGTCGGCAGCGTCAGATGTGTATAAGAGACAGTGACTGACTGCCTATGACG |
| **B3F/B3R** | CAAGCAGAAGACGGCATACGAGATACTGAGCTGTCTCGTGGGCTCGGAGATGTGTATAAGAGACAGGCGTACGTAGACGGCCAGT/AATGATACGGCGACCACCGAGATCTACACGAATTCGCTCGTCGGCAGCGTCAGATGTGTATAAGAGACAGGTGACTGACTGCCTATGACG |
| **B4F/B4R** | CAAGCAGAAGACGGCATACGAGATTTAGGCACGTCTCGTGGGCTCGGAGATGTGTATAAGAGACAGCGTACGTAGACGGCCAGT/AATGATACGGCGACCACCGAGATCTACACAGCTCAGTTCGTCGGCAGCGTCAGATGTGTATAAGAGACAGAGTGACTGACTGCCTATGACG |
| **B5F/B5R** | CAAGCAGAAGACGGCATACGAGATCTCCGATTGTCTCGTGGGCTCGGAGATGTGTATAAGAGACAGATACGTACGTAGACGGCCAGT/AATGATACGGCGACCACCGAGATCTACACGTGCCTAATCGTCGGCAGCGTCAGATGTGTATAAGAGACAGGACGACTGCCTATGACG |
| **B6F/B6R** | CAAGCAGAAGACGGCATACGAGATTTCACAGGGTCTCGTGGGCTCGGAGATGTGTATAAGAGACAGTACGTACGTAGACGGCCAGT/AATGATACGGCGACCACCGAGATCTACACACGTAAGGTCGTCGGCAGCGTCAGATGTGTATAAGAGACAGTGACTGACTGCCTATGACG |
| **B7F/B7R** | CAAGCAGAAGACGGCATACGAGATCAGAGGTAGTCTCGTGGGCTCGGAGATGTGTATAAGAGACAGGCGTACGTAGACGGCCAGT/AATGATACGGCGACCACCGAGATCTACACCCTGTGAATCGTCGGCAGCGTCAGATGTGTATAAGAGACAGGTGACTGACTGCCTATGACG |
| **B8F/B8R** | CAAGCAGAAGACGGCATACGAGATGGCATCATGTCTCGTGGGCTCGGAGATGTGTATAAGAGACAGCGTACGTAGACGGCCAGT/AATGATACGGCGACCACCGAGATCTACACTACCGAGTTCGTCGGCAGCGTCAGATGTGTATAAGAGACAGAGTGACTGACTGCCTATGACG |
| **B9F/B9R** | CAAGCAGAAGACGGCATACGAGATTATGACCGGTCTCGTGGGCTCGGAGATGTGTATAAGAGACAGATACGTACGTAGACGGCCAGT/AATGATACGGCGACCACCGAGATCTACACGGAATGCATCGTCGGCAGCGTCAGATGTGTATAAGAGACAGGACTGACTGCCTATGACG |
| **B10F/B10R** | CAAGCAGAAGACGGCATACGAGATATACGCTGGTCTCGTGGGCTCGGAGATGTGTATAAGAGACAGTACGTACGTAGACGGCCAGT/AATGATACGGCGACCACCGAGATCTACACAATCGGAGTCGTCGGCAGCGTCAGATGTGTATAAGAGACAGTGACTGACTGCCTATGACG |
| **B11F/B11R** | CAAGCAGAAGACGGCATACGAGATACGTTCTCGTCTCGTGGGCTCGGAGATGTGTATAAGAGACAGGCGTACGTAGACGGCCAGT/AATGATACGGCGACCACCGAGATCTACACGAGAACGTTCGTCGGCAGCGTCAGATGTGTATAAGAGACAGGTGACTGACTGCCTATGACG |
| **B12F/B12R** | CAAGCAGAAGACGGCATACGAGATAATTGGCCGTCTCGTGGGCTCGGAGATGTGTATAAGAGACAGCGTACGTAGACGGCCAGT/AATGATACGGCGACCACCGAGATCTACACGGCCAATT TCGTCGGCAGCGTCAGATGTGTATAAGAGACAG AGTGACTGACTGCCTATGACG |
| **B13F/B13R** | CAAGCAGAAGACGGCATACGAGATCATGGCATGTCTCGTGGGCTCGGAGATGTGTATAAGAGACAGATACGTACGTAGACGGCCAGT/AATGATACGGCGACCACCGAGATCTACACTTCGCATCTCGTCGGCAGCGTCAGATGTGTATAAGAGACAGGACTGACTGCCTATGACG |
| **B14F/B14R** | CAAGCAGAAGACGGCATACGAGATACTCGGTAGTCTCGTGGGCTCGGAGATGTGTATAAGAGACAGTACGTACGTAGACGGCCAGT/AATGATACGGCGACCACCGAGATCTACACATGCCATGTCGTCGGCAGCGTCAGATGTGTATAAGAGACAGTGACTGACTGCCTATGACG |
| **B15F/B15R** | CAAGCAGAAGACGGCATACGAGATGAGTTCCAGTCTCGTGGGCTCGGAGATGTGTATAAGAGACAGGCGTACGTAGACGGCCAGT/AATGATACGGCGACCACCGAGATCTACACTGATCACGTCGTCGGCAGCGTCAGATGTGTATAAGAGACAGGTGACTGACTGCCTATGACG |

**qPCR:**

| **F:** GCAACCTGCCCTWTAGCTTG | Ref: Kešnerová et al. (2017). |
| --- | --- |
| **R:** GCCCATCCTKTAGTGACAGC | Ref: Kešnerová et al. (2017). |
