# Supplementary material for: Niche partitioning facilitates coexistence of closely related honey bee gut bacteria
Source: eLife. 2021 Jul 19;10:e68583. doi: 10.7554/eLife.68583 (PMC8456714; doi:10.7554/eLife.68583)
Supplement: Supplementary file 2. [file elife-68583-supp2.docx]

**COG Code COG Function**

J Translation, ribosomal structure and biogenesis

A RNA processing and modification

K Transcription

L Replication, recombination and repair

B Chromatin structure and dynamics

D Cell cycle control, cell division, chromosome partitioning

Y Nuclear structure

V Defense mechanisms

T Signal transduction mechanisms

M Cell wall/membrane/envelope biogenesis

N Cell motility

Z Cytoskeleton

W Extracellular structures

U Intracellular trafficking, secretion, and vesicular transport

O Posttranslational modification, protein turnover, chaperones

X Mobilome: prophages, transposons

C Energy production and conversion

G Carbohydrate transport and metabolism

E Amino acid transport and metabolism

F Nucleotide transport and metabolism

H Coenzyme transport and metabolism

I Lipid transport and metabolism

P Inorganic ion transport and metabolism

Q Secondary metabolites biosynthesis, transport and catabolism

R General function prediction only

S Function unknown
